# Trajectories of cortical surface area and cortical volume maturation in normal brain development

**DOI:** 10.1016/j.dib.2015.10.044

**Published:** 2015-11-10

**Authors:** Simon Ducharme, Matthew D. Albaugh, Tuong-Vi Nguyen, James J. Hudziak, J.M. Mateos-Pérez, Aurelie Labbe, Alan C. Evans, Sherif Karama, for the Brain Development Cooperative Group

**Affiliations:** a Montreal Neurological Institute, McConnell Brain Imaging Centre, McGill University, 3801 University Street, Montreal, QC, Canada H3A 2B4; b McGill University Health Centre, Department of Psychiatry, McGill University, 1025 Pine Avenue West, Montreal, QC, Canada H3A 1A1; c Vermont Centre for Children, Youth and Families, Fletcher Allen Pediatric Psychiatry, University of Vermont, 1 South Prospect Street, Arnold, Level 3, Burlington, VT, USA; d McGill University Health Centre, Department of Obstetrics and Gynecology, McGill University, 1025 Pine Avenue West, Montreal, QC, Canada H3A 1A1; e Douglas Mental Health University Institute, Department of Psychiatry, McGill University, 6875 Lasalle Boulevard, Verdun, QC, Canada H4H 1R3; f Douglas Mental Health University Institute, Department of Epidemiology, Biostatistics and Occupational Health, McGill University, 6875 Lasalle Boulevard, Verdun, QC, Canada H4H 1R3

**Keywords:** Cortical surface area, Cortical volume, Magnetic resonance, imaging, Brain development, Cortical thickness

## Abstract

This is a report of developmental trajectories of cortical surface area and cortical volume in the NIH MRI Study of Normal Brain Development. The quality-controlled sample included 384 individual typically-developing subjects with repeated scanning (1–3 per subject, total scans *n*=753) from 4.9 to 22.3 years of age. The best-fit model (cubic, quadratic, or first-order linear) was identified at each vertex using mixed-effects models, with statistical correction for multiple comparisons using random field theory. Analyses were performed with and without controlling for total brain volume. These data are provided for reference and comparison with other databases. Further discussion and interpretation on cortical developmental trajectories can be found in the associated Ducharme et al.׳s article “Trajectories of cortical thickness maturation in normal brain development – the importance of quality control procedures” (Ducharme et al., 2015) [1].

**Specifications Table**

**Table:** 

Subject area	*Neuroscience*
More specific subject area	*Brain Development*
Type of data	*Figures and description of anatomical findings*
How data was acquired	*All subjects underwent extensive cognitive, neuropsychological and behavioral testing along with up to three MRI brain scans (3D T1-weighted) at two-year intervals.*
Data format	*MRI processed with the Civet pipeline*
Experimental factors	*A visual quality control of all native and post-processing images was applied by two investigators.*
Experimental features	*Each subject׳s absolute native-space local cortical surface area and cortical volume was linearly regressed against age in years at each cortical point using mixed-effects models (diagonal structure). Scanner and gender were included as control variables. Analyses were performed with and without controlling for total brain volume. Whole brain random field theory (RFT) p*≤*0.05 corrections (peaks and clusters) for multiple comparisons were applied for all statistical analyses.*
Data source location	*431 healthy subjects between 4.6 and 18 years of age were recruited throughout 6 sites in the USA, using a population-based sampling method to ensure socio-demographic representation of the American population.*
Data accessibility	*Data from the NIH MRI Study of Normal Brain Development can be publicly accessed through the website*http://pediatricmri.nih.gov/nihpd/info/index.html

## Value of the data

• Data provide a comprehensive vertex-wide description of cortical surface area and cortical volume developmental trajectories in a large sample of healthy children, using only quality controlled data.
• The provided figures and descriptions can be used to compare developmental trajectories with other private and public databases.
• Identified trajectories provide an important comparative baseline for studies of neurodevelopmental abnormalities associated with pediatric neuropsychiatric disorders.

## 1 Data

Data on developmental trajectories for cortical surface area (CSA) and cortical volume (CV) are sequentially presented using a figure format.

### 1.1 Cortical surface area

**Fig. 1:**
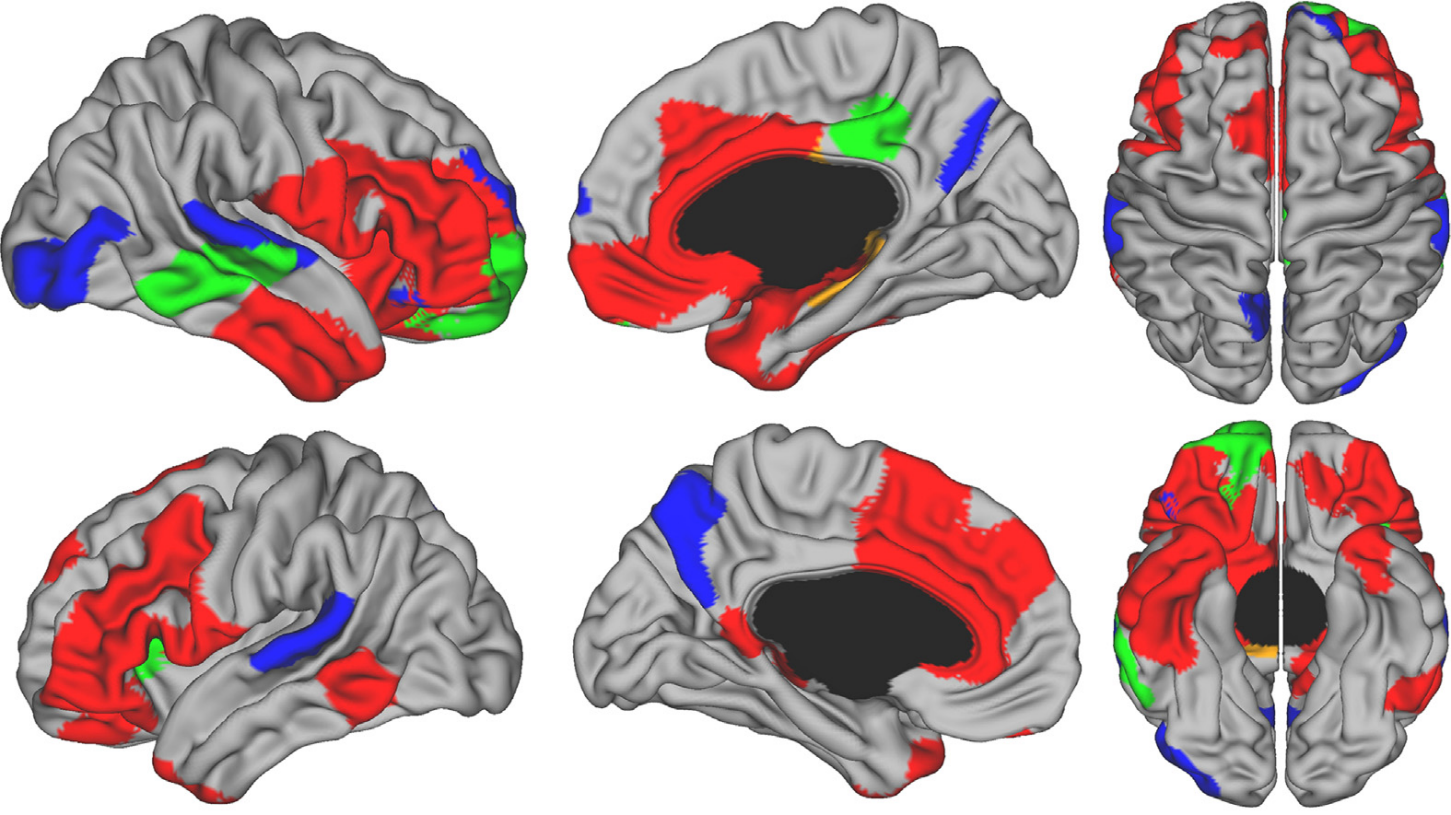
Developmental trajectories of local cortical surface area from 4.9 to 22.3 years of age. Brain areas were the best fitting model is cubic are in green, quadratic in red, and first-order linear in blue. Areas in gray showed no significant changes over time. Controlled for gender and scanner.

**Fig. 2:**
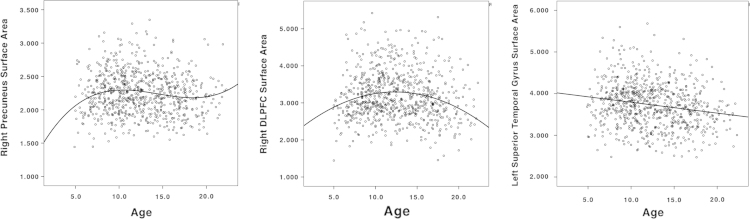
Prototypical scatterplots of mean cortical surface area in brain areas demonstrating a significant cubic (left column – right posterior cingulate cortex), quadratic (middle column – right DLPFC) and first-order linear (right column – left superior temporal gyri) association with age when controlling for gender and scanner.

Fig. 1 illustrates the best fitting developmental trajectories of absolute changes in CSA over time (not controlling for TBV). Cubic trajectories were seen non-symmetrically in the right lateral temporal lobe, right rostral middle frontal cortex, right frontal pole, right orbitofrontal cortex (OFC), right posterior cingulate cortex, and left frontal insula (pars triangularis). Quadratic trajectories were observed diffusely in lateral and medial frontal lobe, including bilateral dorsolateral prefrontal cortex (DLPFC) and anterior cingulate cortex (ACC). There were a few additional areas in bilateral temporal poles and left posterior lateral temporal cortex. First-order linear decline in CSA was limited to a few areas including most notably bilateral superior temporal gyri, bilateral precuneus, and right lateral occipital lobe. Of note, more than 50% of the cortical surface did not show significant change over time in this age range. Fig. 2 illustrates prototypical trajectories in significant clusters. Quadratic areas mainly followed an ‘inverted U’ shape, while cubic areas showed an initial increase of CSA followed small magnitude changes after age 10.

**Fig. 3:**
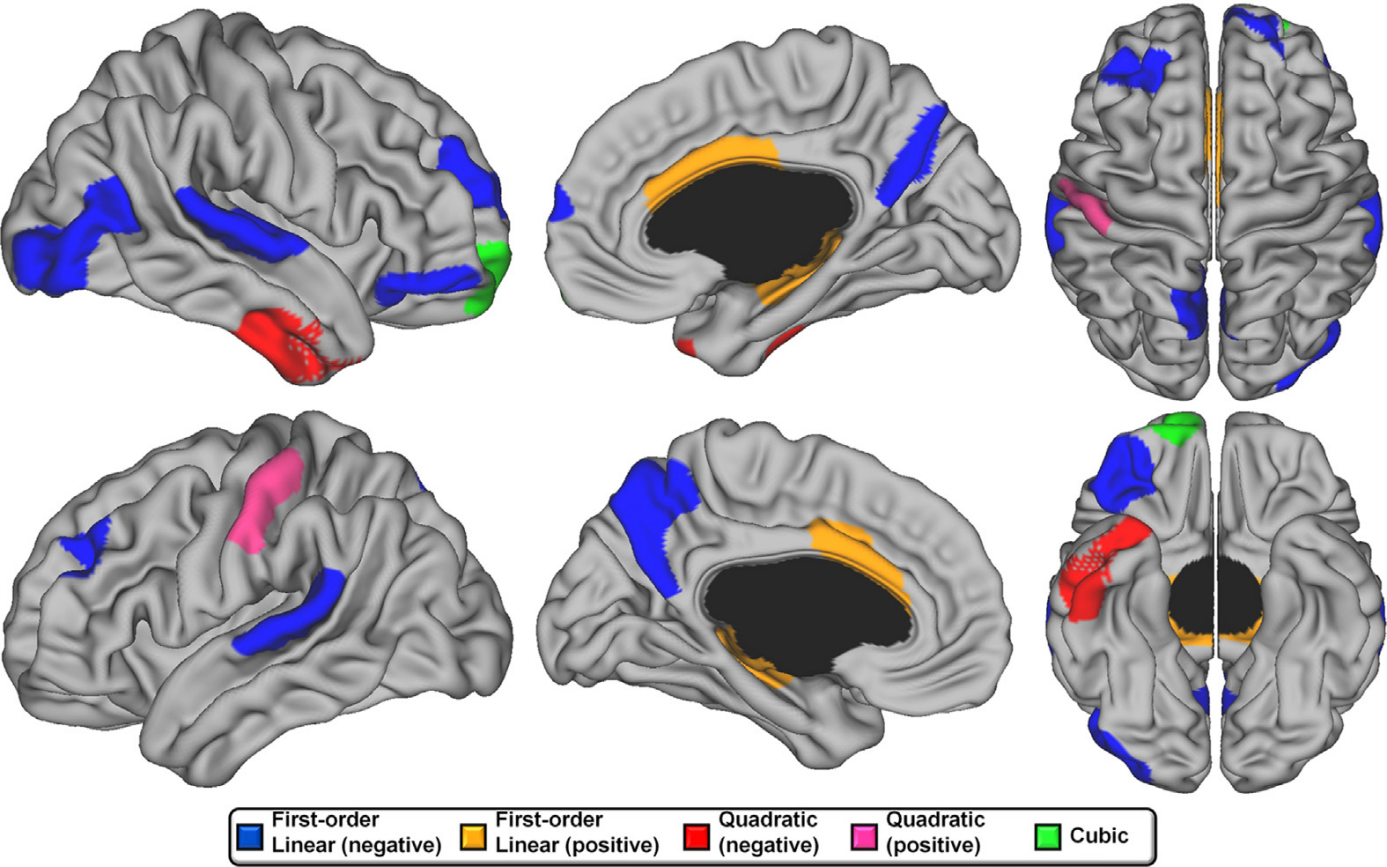
Developmental trajectories of local cortical surface area from 4.9 to 22.3 years of age. Brain areas were the best fitting model is cubic are in green, negative quadratic in red, positive quadratic in pink, negative first-order linear in blue, and positive first-order linear in orange. Areas in gray showed no significant changes over time. Controlled for total brain volume, gender and scanner.

**Fig. 4:**
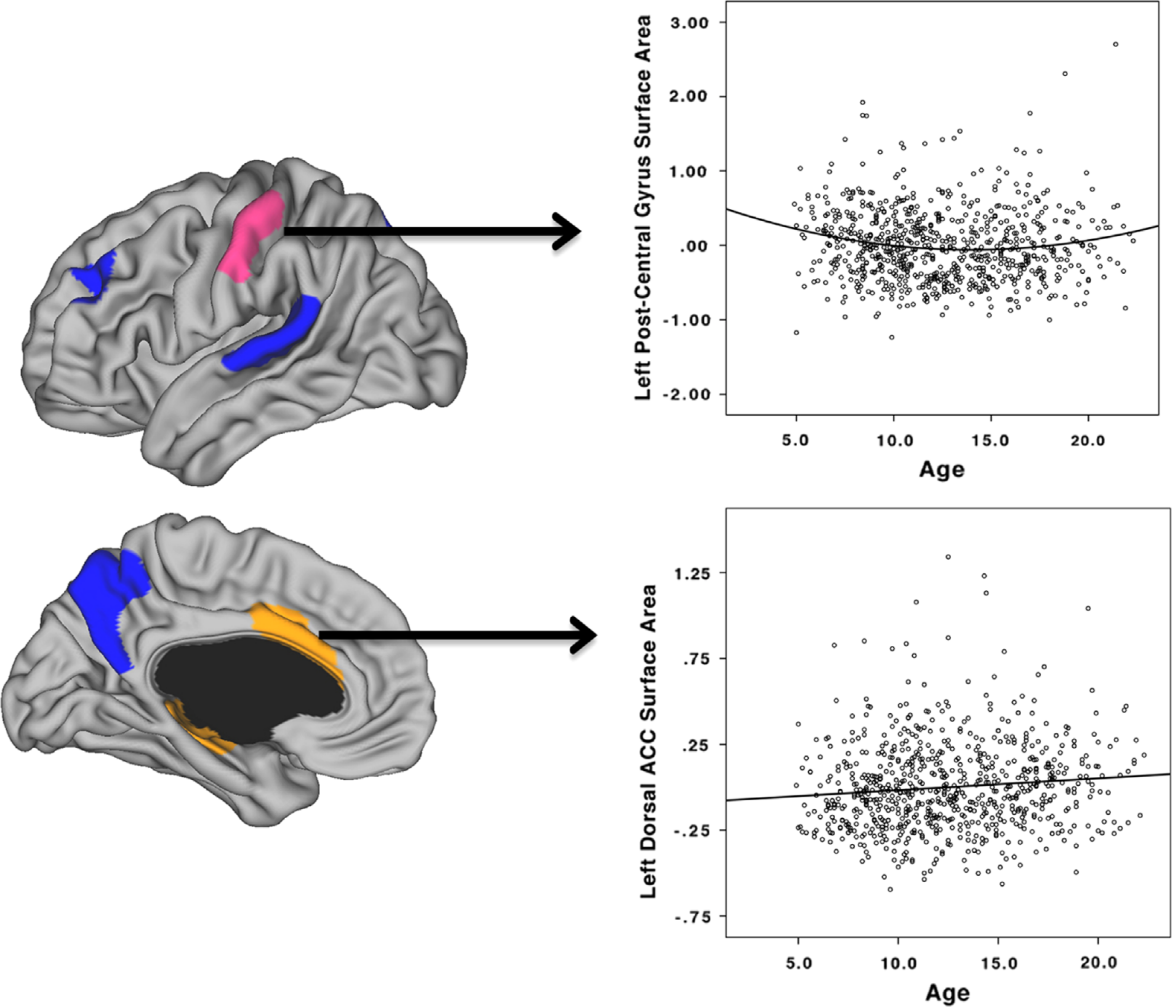
Scatterplots illustrating relative developmental trajectories of the left post-central gyrus (above) and the left anterior cingulate cortex (below). Values on the *Y*-axis represent cortical surface area of the region of interest controlled for total brain volume, scanner, and gender (unstandardized residuals).

Since CSA is strongly linked to TBV, analyses including TBV as a control variable are important to determine relative remodeling over time. These analyses revealed that the great majority of the brain showed no change in CSA between 4.9 and 22.3 years of age (Fig. 3). Small areas of cubic developmental trajectories were found only in the right frontal pole/OFC. Quadratic maturation was found in the right anterior temporal lobe. Monotonic linear decline in CSA was seen in bilateral superior temporal gyri, bilateral middle frontal cortex, bilateral precuneus, and right lateral occipital lobe. Bilateral ACC and parahippocampal gyri showed a relative increase in CSA over time, and the left post-central gyrus showed a relative decrease followed by an increase after 15 years of age (Fig. 4).

### 1.2 Cortical volume

**Fig. 5:**
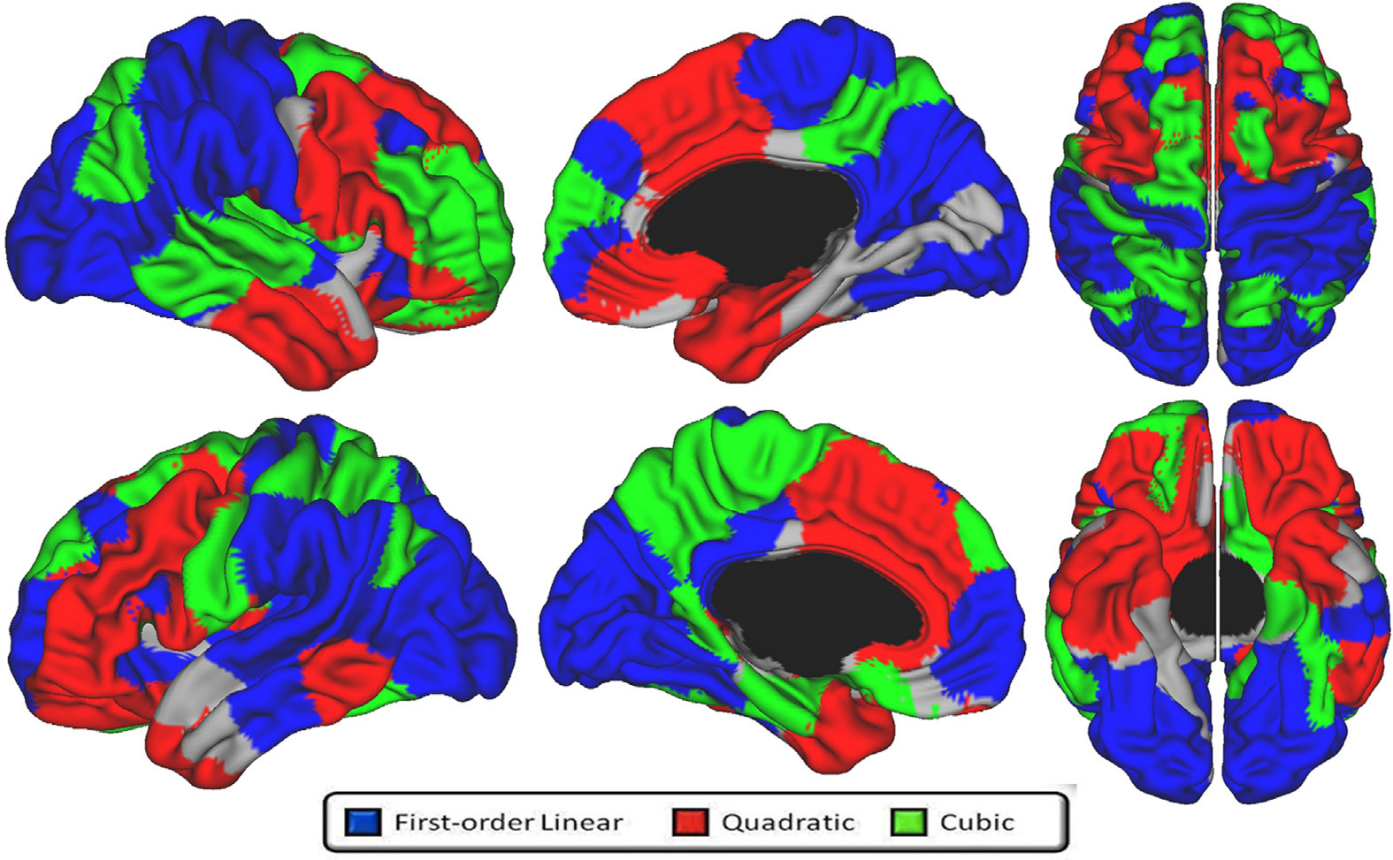
Developmental trajectories of local cortical volume from 4.9 to 22.3 years of age. Brain areas were the best fitting model is cubic are in green, quadratic in red, and first-order linear in blue. Areas in gray showed no significant changes over time. Controlled for gender and scanner.

**Fig. 6:**
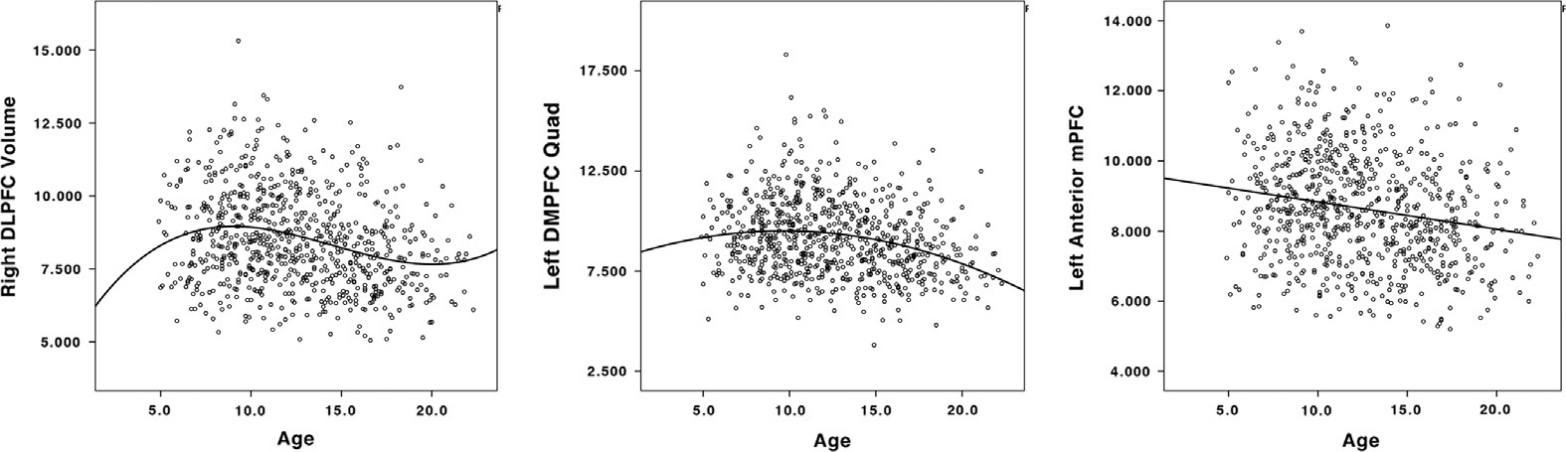
Prototypical scatterplots of mean cortical volume in brain areas demonstrating a significant cubic (left column – right DLPFC), quadratic (middle column – left dmPFC) and first-order linear (right column – left anterior mPFC) association with age when controlling for gender and scanner.

Fig. 5, Fig. 6 illustrate developmental trajectories for absolute CV (without controlling for TBV), which includes the combined impact of cortical thickness (described in the associated article [1]) and CSA. There was a complex interplay of different complexity levels throughout the brain, with a predominance of cubic/quadratic models in the frontal lobes and the anterior temporal lobes, and first-order linear decline in the occipital lobes and many parietal areas (Fig. 5, Fig. 6).

**Fig. 7:**
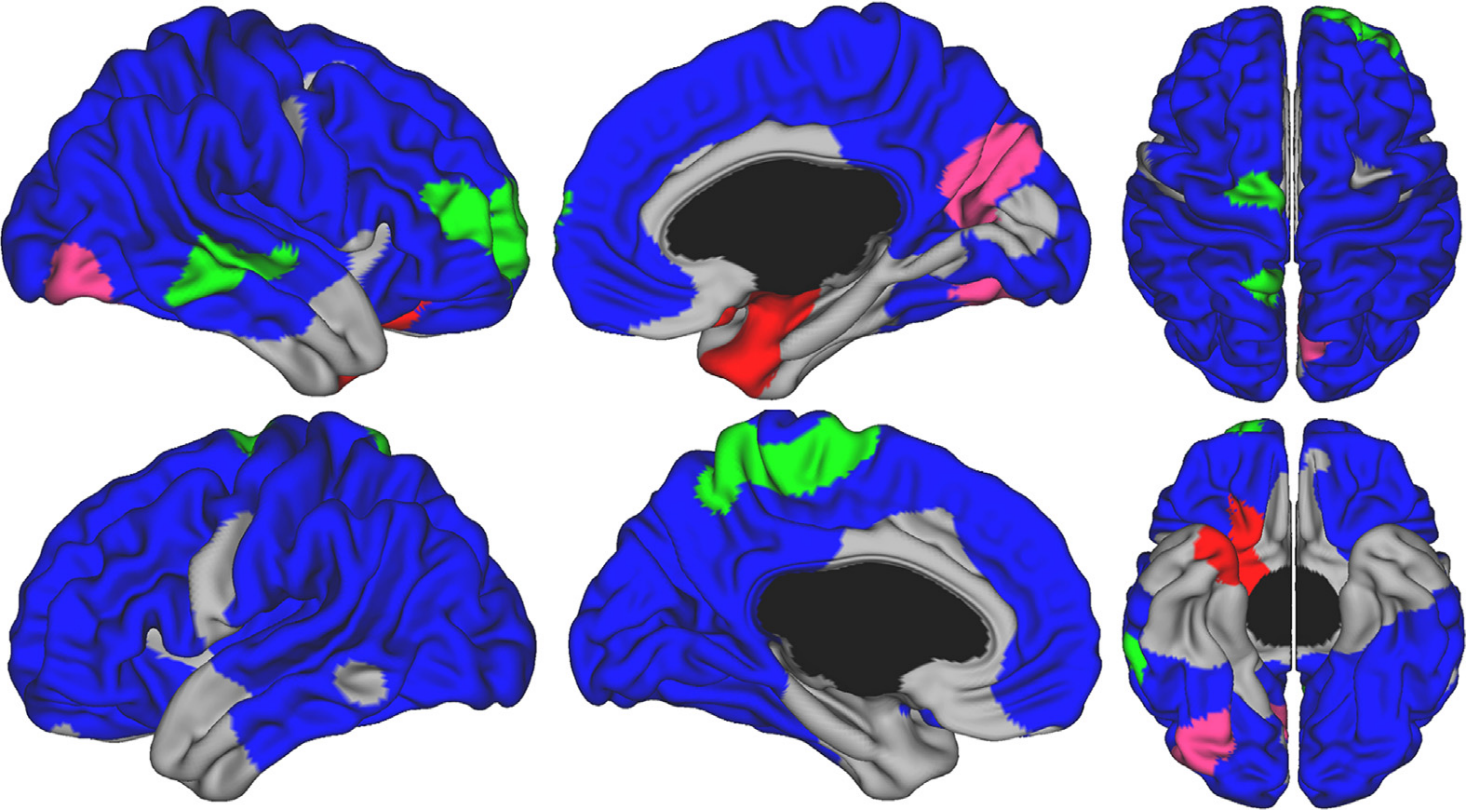
Developmental trajectories of local cortical volume from 4.9 to 22.3 years of age. Brain areas were the best fitting model is cubic are in green, quadratic in red, negative quadratic in pink, and negative first-order linear in blue. Areas in gray showed no significant changes over time. Controlled for total brain volume, gender and scanner.

When controlling for TBV, the great majority of the cortical surface showed a monotonic linear decline in CV over time, reflecting predominantly the impact of cortical thickness (Fig. 7). Notable exceptions included bilateral medial temporal areas, temporal poles, and the ACC, which showed no change over time. For the ACC the absence of change represents the combination of cortical thinning with concurrent expansion of CSA.

## 2 Experimental design, materials and methods

### 2.1 Sampling and recruitment

Subjects were recruited at 6 pediatric study centers across the USA with a population-based sampling method seeking to minimize selection biases [2]. There were extensive exclusion criteria including the presence of a current or past Axis I diagnosis established with the Diagnostic Interview for Children and Adolescents (with the exception of simple phobia, social phobia, adjustment disorder, oppositional defiant disorder, enuresis, encopresis, and nicotine dependence), any Child Behavior Checklist syndrome *T*-score ≥70, family history of major axis 1 disorder, family history of inherited neurological disorder or mental retardation due to non-traumatic events, abnormality on neurological examination, gestational age at birth <37 weeks or >42 weeks, and intra-uterine exposure to substances known or highly suspected to alter brain structure or function. A more exhaustive list of inclusion/exclusion criteria is available in [3].

Based on available US Census 2000 data, 431 healthy children from 4 years and 6 months to 18 years and 3 months (age at the first visit) were recruited (Objective 1) with continuous monitoring in order to ensure that the sample was demographically representative on the basis of variables that included age, gender, ethnicity, and socioeconomic status. Informed consent from parents and child assent were obtained for all subjects.

All subjects underwent extensive cognitive, neuropsychological and behavioral testing along with up to three MRI brain scans at two-year intervals. Structural MRI and clinical/behavioral data were consolidated and analyzed within a purpose-built database at the Data Coordinating Center of the Montreal Neurological Institute (MNI), McGill University.

### 2.2 MRI protocol

Subjects underwent 30–45 min of data acquisition (1.5T), with whole brain coverage and multiple contrasts (T1W, T2W and PDW) [3]. A 3D T1-weighted spoiled gradient recalled echo sequence was selected. The protocol provided 1 mm isotropic data from the entire head, except for subjects scanned on GE scanners for which slice thickness was increased to 1.5 mm due to the limited maximum number of slices. As the priority measure for Objective 1, it was acquired immediately following the localizer scan and, if significant motion artifacts were observed, was immediately repeated. Sagittal acquisition was chosen, being the most efficient way to obtain complete head coverage. For subjects who could not tolerate this optimal procedure, a fallback MR protocol that consisted of shorter 2D acquisitions was used.

Dual contrast, proton density, and T2-weighted (PDW and T2W) acquisitions provided additional information for automated multi-spectral tissue classification/segmentation. An optimized 2D multislice (2 mm) dual echo fast spin echo sequence was used. An oblique axial orientation (parallel to the AC–PC line) was selected. Both American College of Radiology and living phantoms (volunteers repeatedly scanned at each site) were regularly scanned at each site to confirm the inter-site reliability of anatomical measurements [3], [4], [5].

### 2.3 Automated image processing

All quality-controlled MR images were processed through the CIVET pipeline (version 1.1.11) (http://wiki.bic.mni.mcgill.ca/index.php/CIVET) developed at the MNI for fully automated structural image analysis. Detailed steps are described in the associated article. [1]

### 2.4 Visual quality control

Given the sensitivity of automated CTh measurements to small movement artefacts [6], a visual quality control (QC) of each subject׳s extracted white and gray matter surfaces was carried out by two independent investigators to ensure that there were no aberrations for a given subject (inter-rater reliability was 0.93) [7] that could affect CTh estimates. Details on the QC procedure are provided in the associated article. [1]

In total, 753 out of 954 MRI scans (410 females, 343 males) were kept for statistical analyses. This included 384 different subjects from ages 4.9 to 22.3 (age at the last follow-up scan) (mean 12.48±3.9). 101 subjects had three MRI scans, 167 had two MRI scans and 116 had one MRI scan. Figure 1 in the main manuscript [1] shows the age distribution of all data points.

### 2.5 Statistical analyses

Statistical analyses on vertex-wide CSA and CV were implemented using SurfStat (http://www.math.mcgill.ca/keith/surfstat/), a statistical toolbox created for MATLAB (The MathWorks, Inc., Nathan, Massachusetts). Each subject׳s absolute native-space local CSA and CV was linearly regressed against age in years (age in days/365.25) at each cortical point using mixed-effects models (diagonal structure). Mixed-effect models permit the implementation of linear regressions in samples combining subjects with different number of measurements, providing a way in which to analyze unbalanced longitudinal data while maximizing statistical power [8], [9], [10]. In each mixed-effects model, subject ID was entered as a random effect in order to account for within-individual factors. Whole brain random field theory (RFT) *p*≤0.05 corrections (peaks and clusters) for multiple comparisons were applied for all statistical analyses. The age variable was mean centered for all analyses. To avoid confounding factors coming from the multisite nature of this study, scanner number was added as a categorical control variable in the models. There were significant differences in mean age between scanners (12 in total) because recent machines were only used for follow-up visits in which children were older. However, there was no ‘scanner by age’ interaction on mean cortical thickness (ANOVA *F*(11,741)=0.677; *p*=0.761). To facilitate comparison with other studies, terminology from the Desikan surface atlas was used to describe anatomical location of findings in this manuscript [11].

In order to determine the best-fit model for the developmental trajectory of CSA and CV, the most complex cubic model was tested first:

$$Y=intercept+b_{1}Sex+b_{2}Scanner+b_{3}Age+b_{4}Age^{2}+b_{5}Age^{3}+random\left(Subject\_ID\right)+error$$

In a second step, a quadratic model of development was tested to evaluate vertices that did not show a statistically significant cubic trajectory:

$$Y=intercept+b_{1}Sex+b_{2}Scanner+b_{3}Age+b_{4}Age^{2}+random\left(Subject\_ID\right)+error$$

In a third step, a first-order linear regression was implemented to evaluate brain areas that were not significant in cubic or quadratic models:

$$Y=intercept+b_{1}Sex+b_{2}Scanner+b_{3}Age+random\left(Subject\_ID\right)+error$$

It is well known that there is significant remodeling within the developing brain with different changes in cortical, subcortical, white matter and gray matter [12]. To account for these relative changes, the above-described analyses were repeated adding total brain volume (TBV) as a covariate in the model.

## Disclaimer

The views herein do not necessarily represent the official views of the National Institute of Child Health and Human Development, the National Institute on Drug Abuse, the National Institute of Mental Health, the National Institute of Neurological Disorders and Stroke, the National Institutes of Health, the U.S. Department of Health and Human Services, or any other agency of the United States Government.

## Brain Development Cooperative Group

Key personnel from the six pediatric study centers are as follows: **Children**׳s **Hospital Medical Center of Cincinnati**, Principal Investigator William S. Ball, M.D., Investigators Anna Weber Byars, Ph.D., Mark Schapiro, M.D., Wendy Bommer, R.N., April Carr, B.S., April German, B.A., Scott Dunn, R.T.; **Children**׳s **Hospital Boston**, Principal Investigator Michael J. Rivkin, M.D., Investigators Deborah Waber, Ph.D., Robert Mulkern, Ph.D., Sridhar Vajapeyam, Ph.D., Abigail Chiverton, B.A., Peter Davis, B.S., Julie Koo, B.S., Jacki Marmor, M.A., Christine Mrakotsky, Ph.D., M.A., Richard Robertson, M.D., Gloria McAnulty, Ph.D; **University of Texas Health Science Center at Houston**, Principal Investigators Michael E. Brandt, Ph.D., Jack M. Fletcher, Ph.D., Larry A. Kramer, M.D., Investigators Grace Yang, M.Ed., Cara McCormack, B.S., Kathleen M. Hebert, M.A., Hilda Volero, M.D.; **Washington University in St. Louis**, Principal Investigators Kelly Botteron, M.D., Robert C. McKinstry, M.D., Ph.D., Investigators William Warren, Tomoyuki Nishino, M.S., C. Robert Almli, Ph.D., Richard Todd, Ph.D., M.D., John Constantino, M.D.; **University of California Los Angeles**, Principal Investigator James T. McCracken, M.D., Investigators Jennifer Levitt, M.D., Jeffrey Alger, Ph.D., Joseph O’Neil, Ph.D., Arthur Toga, Ph.D., Robert Asarnow, Ph.D., David Fadale, B.A., Laura Heinichen, B.A., Cedric Ireland B.A.; **Children**׳s **Hospital of Philadelphia**, Principal Investigators Dah-Jyuu Wang, Ph.D. and Edward Moss, Ph.D., Investigators Robert A. Zimmerman, M.D., and Research Staff Brooke Bintliff, B.S., Ruth Bradford, Janice Newman, M.B.A. The Principal Investigator of the data coordinating center at **McGill University** is Alan C. Evans, Ph.D., Investigators Rozalia Arnaoutelis, B.S., G. Bruce Pike, Ph.D., D. Louis Collins, Ph.D., Gabriel Leonard, Ph.D., Tomas Paus, M.D., Alex Zijdenbos, Ph.D., and Research Staff Samir Das, B.S., Vladimir Fonov, Ph.D., Luke Fu, B.S., Jonathan Harlap, Ilana Leppert, B.E., Denise Milovan, M.A., Dario Vins, B.C., and at **Georgetown University**, Thomas Zeffiro, M.D., Ph.D. and John Van Meter, Ph.D. Ph.D. Investigators at the Neurostatistics Laboratory, **Harvard University/McLean Hospital,** Nicholas Lange, Sc.D., and Michael P. Froimowitz, M.S., work with data coordinating center staff and all other team members on biostatistical study design and data analyses. The Principal Investigator of the Clinical Coordinating Center at **Washington University** is Kelly Botteron, M.D., Investigators C. Robert Almli Ph.D., Cheryl Rainey, B.S., Stan Henderson M.S., Tomoyuki Nishino, M.S., William Warren, Jennifer L. Edwards M.SW., Diane Dubois R.N., Karla Smith, Tish Singer and Aaron A. Wilber, M.S. The Principal Investigator of the Diffusion Tensor Processing Center at the **National Institutes of Health** is **Carlo Pierpaoli, MD, Ph.D.,** Investigators **Peter J. Basser, Ph.D., Lin-Ching Chang, Sc.D.,** Chen Guan Koay**, Ph.D. and Lindsay Walker, M.S.** The Principal Collaborators at the **National Institutes of Health** are Lisa Freund, Ph.D. (NICHD), Judith Rumsey, Ph.D. (NIMH), Lauren Baskir, Ph.D. (NIMH), Laurence Stanford, PhD. (NIDA), Karen Sirocco, Ph.D. (NIDA) and from NINDS, Katrina Gwinn-Hardy, M.D., and Giovanna Spinella, M.D. The Principal Investigator of the Spectroscopy Processing Center at the **University of California Los Angeles** is **James T. McCracken, M.D., Investigators** Jeffry R. Alger, Ph.D., Jennifer Levitt, M.D., Joseph O׳Neill, Ph.D.
